# Association of the genetic polymorphisms of the *ACE *gene and the *eNOS *gene with lupus nephropathy in northern Chinese population

**DOI:** 10.1186/1471-2350-11-94

**Published:** 2010-06-14

**Authors:** X Li, J An, R Guo, Z Jin, Y Li, Y Zhao, F Lu, H Lian, P Liu, Y Zhao, X Jin

**Affiliations:** 1 Department of Rheumatology, the Second Affiliated Hospital of Harbin Medical University, Harbin, 150081, PR China; 2 Department of Pathology, Harbin Medical University, Harbin, 150081, PR China; 3 Chinese medical hospital of Weihai city, Weihai, 264200, PR China; 4 Department of Pathology, Mudanjiang Medical University, Mudanjiang, 157011, PR China; 5 Heilongjiang University of Chinese Medicine, Harbin, 150040, PR China; 6 Department of laboratory Diagnosis, the First Affiliated Hospital of Harbin Medical University, Harbin, 150001, PR China; 7 Department of Rheumatology,the First affiliated hospital of Baotou Medical College, Baotou, 014010, PR China; 8 Laboratory of Medical Genetics, Harbin Medical University, Harbin, 150081, PR China

## Abstract

**Background:**

It has been reported that some single nucleotide polymorphisms (SNPs) of the angiotensin converting enzyme (*ACE*) gene and the endothelial nitric oxide synthase (*eNOS*) gene are associated with the development of systemic lupus erythematosus (SLE) and the progression of nephropathy. The aim of this study was to evaluate the possible association between six SNPs (*A-5466C*, *T-3892C*, *A-240T*, *C1237T*, *G2215A *and *A2350G*) of the *ACE *gene and two SNPs (*T-786C *and *G894T*) of the *eNOS *gene with lupus nephropathy in a northern Chinese population.

**Methods:**

In this study, 225 patients with lupus nephropathy were compared to 232 healthy controls, matched by gender, age and ethnicity. Following the extraction of genomic DNA from the leukocytes in the peripheral blood, the genotypes of the eight selected SNPs were determined by the method of PCR-RFLP; the haplotypes were inferred using PHASE 2.1. The associations between the SNPs and the risk of lupus nephropathy were analyzed using Chi-square test and Logistic regression with SPSS13.0 software.

**Results:**

Statistically significant differences of the allele frequency distribution of three SNPs (*A-5466C*, *A2350G *and *G894T*) were observed between cases and controls (*P *< 0.05). Among the 53 haplotypes identified, the frequencies of five haplotypes (CTTCGA, ACTTAA, ACATGG, ACACGG and ATTCGA) were significantly different between cases and controls (*P *< 0.05).

**Conclusions:**

Our study indicated an association between the risk of lupus nephropathy and the sequence variations of both the *ACE *gene and the *eNOS *gene, which may play an important role in the pathogenesis of lupus nephropathy in the northern Chinese population. Further studies are warranted to validate our findings.

## Background

Systemic lupus erythematosus (SLE) is a complex autoimmune disease involving environmental, genetic and hormonal elements [1-3]; also it is a multisystem disease with a variable course and a wide range of clinical manifestations[4], such as lupus nephropathy. Renal injury in SLE is one of the most serious complications and its pathogenesis has not yet been completely clarified [1].

It has been extensively documented that genetic factors play an important role in the development and progression of both SLE and lupus nephropathy [5-7]. Many studies have showed the critical role of the SNPs of the *ACE *gene and the *eNOS *gene in the process of the occurrence and progression of SLE [8-10].

As is widely known that the reninangiotensin system (RAS) is usually involved in the progression of renal diseases [11]. ACE affects various clinical manifestations through the reninangiotensin system by promoting the formation of angiotensin II and inactivating bradykinin [2]. In human, the *ACE *gene is located on chromosome 17q22-q24 [12] and is expressed in a wide range of tissues, such as lung, vascular endothelium, kidney, heart and testes [9]. Many studies, including one study in a Chinese population [13], have suggested that a 250 bp insertion/deletion (I/D) polymorphism of the *ACE *gene was associated with SLE and renal injury [1,13]. Recent studies have reported that several SNPs (*A-5466C*, *T-3892C*, *A-240T*, *CJ237T*, *G2215A *and *A2350G*) of the *ACE *gene may affect the risk of certain autoimmune diseases such as essential hypertension, left ventricular hypertrophy, IgA nephropathy, diabetic nephropathy and so on [14-16]. Therefore, we presume that these SNPs of the *ACE *gene may also play an important role in the molecular mechanisms of lupus nephropathy.

Nitric oxide (NO) plays an important role in the pathogenesis of SLE, with an elevated level of expression in SLE patients than in healthy controls [17]. NO synthesis is tightly regulated by nitric oxide synthases (NOS), the latter has three isoforms: neuronal (nNOS), inducible (iNOS) and endothelial (eNOS) [18]. The *eNOS *gene is located on chromosome 7q35-q36 [19], which is an important factor in the process of immunity reaction and the production of NO. It has been reported that two SNPs (*T-786C *and *G894T*) of the *eNOS *gene were associated with the susceptibility of vascular, infectious and autoimmune diseases, such as resistant hypertension, ischemic stroke, essential hypertension, and lacunar infarction [20-22]. These SNPs may alter the level of the *eNOS *gene expression or change the protein product of the gene, and is relevant either to the pathogenesis of SLE or the progression of specific manifestations of diseases such as atherosclerosis and renal complications [23,24]. Therefore, the *eNOS *gene is a candidate gene for the analysis of SLE susceptibility [8,25].

However, the associations between the above mentioned SNPs and the risk of lupus nephropathy have not been evaluated in the Chinese population. The aim of the present study is to investigate the association of these SNPs with lupus nephropathy in a northern Chinese population.

## Methods

### Subjects

Totally 225 lupus nephropathy patients were recruited from the department of rheumatology, the Second Affiliated Hospital of Harbin Medical University, located in northern China. All patients were classified as lupus nephropathy according to the American College of Rheumatology (ACR) renal criteria [26,27] or the evidence of the renal biopsy for lupus nephropathy. The methods used for the determination of urinalysis and blood pressure, as well as the diagnostic criteria for serum creatinine and proteinuria were following the World Health Organization (WHO) criteria[28].

The control subjects were enrolled from the individuals who underwent a health examination in the Second Affiliated Hospital of Harbin Medical University. They had no history of rheumatism and immunology diseases, nor any abnormal clinical examination results. Both cases and controls were matched by gender, age and ethnicity. All study participants provided informed consent and donated peripheral blood. This study was approved by the appropriate local authority. The major characteristics of the study subjects were listed in Table 1.

**Table 1:** Clinical and laboratory characteristics of healthy controls and lupus nephropathy

Characteristics	Control	Lupus nephropathy
n	232	225
Mean age (years) mean ± SD	34.9 ± 9.8	35.2 ± 10.1
Sex ratio (Female: Male)	85/147	81/144
24-hour proteinuria (mg)	-	> 500
persistent hematuria	-	yes
s-Cr(mg/dL)	-	> 1.3

Using the standard method of phenol-chloroform extraction, the DNA samples were prepared from peripheral blood samples anti-coagulated with ACD.

### Genotyping of the SNPs of the *ACE *gene and the *eNOS *gene

The information of the six SNPs of the *ACE *gene and two SNPs of the *eNOS *gene were shown in Table 2, as well as the details of the primers, restriction enzymes and the length of digested fragments. The PCR reactions were performed in a final volume of 25 μl, containing approximately 50 ng genomic DNA, 0.5 μM each primer, 0.2 mM dNTP, 2.0 mM MgCl2, 50 mM KCl, 10 mM Tris HCl (pH 8.4) and 0.2 U Taq DNA Polymerase (TaKaRa). All reactions were performed using a Perkin Elmer 9700. The PCR products were digested and then separated by 2-3% agarose gel electrophoresis.

**Table 2:** The information of the primers and restriction enzymes

SNP	Location	Primer sequence	Enzyme	Amplification length	The length of digested fragments
		F-gccatgtcacatgtattatagga	*EcoRV*	133 bp	24 bp + 109 bp
*ACE A-5466C*	5'UTR				
		R-cgtctttggaaacttgtctgc			
		F-atagtgtatatagggcttggtac	*PstI*	114 bp	90 bp + 24 bp
*ACE T-3892C*	5'UTR				
		R-agaagatatttgcaaagtatgtactg			
		F-tgtcactccggaggcgggaggct	*XbaI*	168 bp	145 bp + 23 bp
*ACE A-240T*	5'UTR				
		R-gagaaagggcctcctctctct			
		F-agtgcacacgggtcacgatg	*BsmBI*	287 bp	95 bp +192 bp
*ACE C1237T*	exon8				
		R-caccaagtagccaaagggcag			
		F-cacaccctgaagtacggcac	*HaeII*	131 bp	109 bp + 22 bp
*ACE G2215A*	exon14				
		R-tcctccagctcctgggcag			
		F-ctgacgaatgtgatggccgc	*BstUI*	122 bp	103 bp + 19 bp
*ACE A2350G*	exon16				
		R-ttgatgagttccacgtatttcg			
		F-tggagatgatggtgtacccca	*MspI*	180 bp	94 bp +46 bp +42 bp
*eNOS T-786C*	5'UTR				
		R-gcctccacccccaccctgtc			
		*F-aaggcaggagacagtggatgga*	*Ban II*	248 bp	163 bp + 85 bp
*eNOS G894T*	Exon7				
		R- cccagtcaatccctttggtgctca			

### Statistical analysis

After the genotypes of each individual were determined, genotype and allele frequencies were calculated by direct counting. Deviations from Hardy-Weinberg equilibrium (HWE) were tested for all SNPs in control subjects. As these loci of the *ACE *gene were tightly linked in a LD block, we conducted a haplotype analysis using PHASE 2.1 software. The associations between the allele frequencies and genotype frequencies and lupus nephropathy were analyzed by the Chi-square test and Logistic regression using SPSS13.0 software for Windows. The relative risk of minor alleles compared with major alleles was estimated using odds ratio (OR) and 95% confidence interval (CI), P-values less than 0.05 were considered statistically significant.

## Results

### Genotype distribution of the *ACE *gene between patients with lupus nephropathy and controls

All loci were in Hardy-Weinberg equilibrium in controls (*p *> 0.05). The allele frequencies of the six SNPs (*A-5466C*, *T-3892C*, *A-240T*, *C1237T*, *G2215A *and *A2350G*) of the *ACE *gene were 0.318 (*-5466C*), 0.411 (*-3892T*), 0.402 (*-240T*), 0.298 (*1237C*), 0.387 (*2215A*) and 0.158 (*2350G*) in cases, and 0.429 (*-5466C*), 0.457 (*-3892T*), 0.379 (*-240T*), 0.295 (*1237C*), 0.358 (*2215A*) and 0.244 (*2350G*) in controls (Table 3).

**Table 3:** Allele frequencies and genotype frequencies of the SNPs of the ACE gene and the eNOS gene in cases and controls

SNP	Genotype	Control N = 232 (%)	Lupus nephropathy N = 225 (%)	*p*	OR (95%CI)
	AA	79(34.05)	108(48.44)	0.002*	***0.559(0.384-0.816)***
	AC	107(46.12)	91(40.44)	0.221	1.260(0.870-1.826)
*ACEA-5466C*	CC	46(19.83)	26(11.56)	0.015*	***1.893(1.124-3.186)***
	A allele	265(57.11%)	307(68.22%)	0.001*	***0.620(0.473-0.813)***
	C allele	199(42.89%)	143(31.78%)	0.001*	***1.612(1.230-2.113)***
	TT	50(21.55)	43(19.11)	0.517	1.163(0.737-1.835)
	TC	112(48.28)	99(44.00)	0.359	1.188(0.822-1.717)
*ACE T-3892C*	CC	70(30.17)	83(36.89)	0.128	0.739(0.501-1.092)
	T allele	212(45.69%)	185(41.11%)	0.163	1.205(0.927-1.566)
	C allele	252(54.31%)	265(58.89%)	0.163	0.830(0.639-1.078)
	AA	93(40.09)	79(35.11)	0.272	1.236(0.846-1.807)
	AT	102(43.97)	111(47.84)	0.250	0.806(0.558-1.164)
*ACEA-240T*	TT	37(15.95)	35(15.56)	0.908	1.030(0.623-1.704)
	A allele	273 (62.07%)	269(59.78%)	0.772	0.962(0.739-1.252)
	T allele	191(37.93%)	181(40.22%)	0.772	1.040(0.799-1.354)
*ACE C1237T*	CC	24(10.34)	19(8.44)	0.487	1.251(0.665-2.353)
	CT	89(38.36)	96(42.67)	0.349	0.836(0.575-1.216)
	TT	119(51.29)	110(48.89)	0.607	1.101(0.763-1.589)
	C allele	137(29.53%)	134(29.78%)	0.934	0.988(0.744-1.312)
	T allele	327(70.47%)	316(70.22%)	0.934	1.012(0.762-1.344)
	GG	98(42.24)	87(38.67)	0.436	1.160(0.798-1.686)
	AG	102(43.97)	102(45.33)	0.769	0.946(0.654-1.368)
*ACEG2215A*	AA	32(13.79)	36(16.00)	0.507	0.840(0.501-1.407)
	G allele	298(64.22%)	276(61.33%)	0.366	1.132(0.865-1.480)
	A allele	166(35.78%)	174(38.67%)	0.366	0.884(0.676-1.156)
	AA	138(59.48)	163(72.44)	0.003*	***0.558(0.377-0.827)***
	AG	75(32.33)	53(23.56)	0.037*	***1.550(1.026-2.343)***
*ACEA2350G*	GG	19 (8.19)	9(4.00)	0.062	2.141(0.947-4.838)
	A allele	351(75.65%)	379(84.22%)	0.001*	***0.582(0.418-0.810)***
	G allele	113(24.35%)	71(15.78%)	0.001*	***1.719(1.235-2.391)***
	CC	9(3.88)	11(4.89)	0.598	0.785(0.319-1.933)
	TC	59(25.43)	57(25.33)	0.981	1.005(0.660-1.532)
*eNOS T-786C*	TT	164(70.69)	157(69.78)	0.831	1.045(0.699-1.560)
	T allele	387(83.41%)	371(82.44%)	0.700	1.070(0.758-1.511)
	C allele	77(16.59%)	79(17.56%)	0.700	0.934(0.662-1.319)
*eNOS G894T*	GG	179(77.16)	195(86.67)	0.008*	***0.520(0.318-0.850)***
	GT	46(19.83)	27(12.00)	0.022*	***1.814(1.083-3.037)***
	TT	7(3.02)	3(1.33)	0.215	2.312(0.593-9.019)
	G allele	404(87.07%)	417(92.67%)	0.005*	***0.533(0.341-0.833)***
	T allele	60(12.93%)	33(7.33%)	0.005*	***1.877(1.201-2.932)***

* *p *< 0.05

There was a significant difference in the frequency of the *-5466C *allele between cases and controls (P = 0.001; OR = 1.612, 95%CI = 1.230-2.113). Similarly, the association between the *2350G *allele and the risk of lupus nephropathy was significant (P = 0.001; OR = 1.719, 95%CI = 1.235-2.391). However, there was no significant association between the other four SNPs (*T-3892C, A-240T, C1237T *and *G2215A*) and lupus nephropathy in this sample (*P *> 0.05; Table 3).

As shown in Table 3, significant associations was observed between the frequencies of the genotypes of *-5466AA/-5466AC+-5466CC *and the risk of lupus nephropathy (*P *= 0.002; OR = 0.559, 95%CI = 0.384-0.816). Such association were also seen between the genotypes of *2350AA/2350AG+2350GG *and lupus nephropathy (*P *= 0.003; OR = 0.558, 95%CI = 0.377-0.827).

Fifty-three haplotypes were identified in these samples and the frequencies of twenty-four haplotypes among them were listed in Table 4. The most common haplotype in lupus nephropathy patients was ACATGA, followed by ACATAA, CCATAA, and CCATGA and so on. Among them, the frequency of the CTTCGA haplotype in lupus nephropathy patients (2.89%) was higher than in healthy controls (0.22%, *P *= 0.001). The associations between other four haplotypes (ACTTAA, ACATGG, ACACGG and ATTCGA) and lupus nephropathy were also significant (*P *< 0.05). However, there were no association between the other haplotypes and lupus nephropathy (*P *> 0.05).

**Table 4:** Haplotype frequencies of the ACE gene in cases and controls

Haplotype	Control 2N = 464 (%)	Lupus nephropathy 2N = 450 (%)	*p*	OR (95%CI)
ACATGA	52(11.21)	49(10.89)	0.878	1.033(0.683-1.562)
CCATAA	23(4.96)	30(6.67)	0.269	0.730(0.417-1.277)
ACATAA	21(4.53)	31(6.89)	0.123	0.641(0.362-1.133)
CCATGA	22(4.74)	25(5.56)	0.577	0.846(0.470-1.524)
ATTCGA	12(2.59)	23(5.11)	0.047*	***0.493(0.242-1.003)***
ACTTGA	22(4.74)	20(4.44)	0.830	1.070(0.576-1.989)
ATATGA	25(5.39)	13(2.89)	0.058	1.914(0.967-3.791)
CCTTAA	32(6.90)	23(5.11)	0.256	1.375(0.792-2.389)
ATTTAA	13(2.80)	21(4.67)	0.136	0.589(0.291-1.191)
ACACGA	13(2.80)	17(3.78)	0.408	0.734(0.352-1.530)
ATATAA	6(1.29)	14(3.11)	0.060	0.408(0.155-1.071)
CCATGG	16(3.45)	11(2.44)	0.370	1.425(0.654-3.106)
ATACGA	11(2.37)	16(3.56)	0.290	0.659(0.302-1.435)
ACTTAA	20(4.31)	6(1.33)	0.007*	***3.333(1.326-8.379)***
ATTTGA	16(3.45)	10(2.22)	0.265	1.571(0.705-3.501)
CCTTAG	6(1.29)	14(3.11)	0.060	0.408(0.155-1.071)
CTTCGA	1(0.22)	13(2.89)	0.001*	***0.073(0.009-0.557)***
ACACGG	11(2.37)	3(0.67)	0.036*	***3.618(1.003-13.056)***
ACTCGA	5(1.08)	9(2.00)	0.256	0.534(0.177-1.605)
ACATGG	11(2.37)	3(0.67)	0.036*	***3.618(1.003-13.056)***
CCACGA	10(2.16)	4(0.89)	0.119	2.456(0.765-7.888)
CCATAG	6(1.29)	6(1.33)	0.957	0.969(0.310-3.028)
ACACAA	3(0.65)	9(2.00)	0.072	0.319(0.086-1.186)
ACTCAA	7(1.51)	10(2.22)	0.425	0.674(0.254-1.786)

* *p *< 0.05

### Genotype distribution of the *eNOS *gene between patients with lupus nephropathy and controls

All loci were in Hardy-Weinberg equilibrium in controls (*P *> 0.05). The allele frequencies were 0.176 (*-786C*) and 0.073 (*894T*) in cases, and 0.166 (*-786C*) and 0.129 (*894T*) in controls (Table 3). There was a significant difference in the allelic frequency of the *894T *allele between cases and controls (*P *= 0.005; OR = 1.877, 95%CI = 1.201-2.932), while the association was not significant between the genotype frequency of the *-786C *and lupus nephropathy (*P *= 0.700; OR = 0.934, 95%CI = 0.662-1.319). As shown in Table 3, the association between the *894GG/894GT+894TT *genotypes and lupus nephropathy was statistically significant (*P *= 0.008; OR = 0.520, 95%CI = 0.318-0.850).

Since both the *ACE *gene and the *eNOS *gene were associated with the risk of lupus nephropathy, we evaluated the interaction of the two genes, using SNPs that were significantly associated with the disease. Neither the interaction of *ACE *A-5466C and *eNOS *G-894T nor the interaction of *ACE *A-2350G and *eNOS *G-894T were significant, *P *values were 0.093 and 0.950, respectively.

## Discussion

Lupus nephropathy happens at a high frequency in patients with SLE, and it has been suggested to be related to abnormal regulation of the complex system consisting of the RAS and the NO system, both regulating vascular tone and inflammation. These two systems may have a profound effect in the pathogenesis and progression of SLE and the development of lupus nephropathy. Multiple genetic and environmental factors may be implicated in the evolution of lupus nephropathy. Different genetic variants contribute to the SLE phenotype from populations of different genetic backgrounds are becoming increasingly apparent [29]. In recent years, complete genome scans have tried to search for SLE susceptibility loci [30], two candidate genes identified are the *ACE *gene and the *eNOS *gene [31], which are inter-related [32]. ACE is a key component of RAS and it converts angiotensin I to II [33]. It inhibits the progression pace of the majority of chronic nephropathies [34]. eNOS is an important isoform of NOS, which plays an important role in autoimmune diseases [23,35]. Polymorphisms of the *eNOS *gene are relevant to the pathogenesis of certain diseases correlate with SLE [36]. As a family-based association analysis has already shown an association between *ACE G-261T *and SLE in a Chinese population [37], we did not test this locus in our study. Six SNPs (*A-5466C*, *T-3892C*, *A-240T*, *C1237T*, *G2215A *and *A2350G*) of the *ACE *gene and two SNPs (*T-786C *and *G894T*) of the *eNOS *gene have been selected since they may affect the susceptibility to some autoimmune diseases [36].

In this study, significant differences in genotype distribution of two SNPs (*A-5466C *and *A2350G*) of the *ACE *gene were observed between cases and controls. The differences are similar to previous reports [38,39], and are strong predictors of plasma ACE levels [40,41]. No association was observed between the allelic frequency distribution of other SNPs and lupus nephropathy, although they have been studied extensively in high prevalence of renal disorders among hypertensive [42,43]. Our results suggested that *ACE -5466C *and *ACE 2350G *allele play a significant protect role in the pathology of renal disease, and *ACE -5466A *allele in homozygote individuals and *ACE 2350A *allele in dominant genetic model were risk allele for lupus nephropathy.

The association between some of the haplotypes and lupus nephropathy were shown to be significant. Among them, the haplotypes ACTTAA, ACACGG and ACATGG play protective role in the renal injury in SLE, while haplotypes ATTCGA and CTTCGA were risk factors. To the best of our knowledge, this is the first study focusing on the SNPs of *ACE *gene and *eNOS *gene in Chinese population.

NO is an important biological molecule, which has a critical role in many biological systems. It has been identified as a potent mediator of immune and inflammatory response. Studies have shown that the levels of NO are significantly elevated in SLE patients in comparison with controls. A correlation has been found between serum NO and SLE disease activity[17]. For example, the *T-786C *and its interaction with *G894T *have impact on the basal nitric oxide activity of renal circulation, essential hypertension and diabetic nephropathy in multi-ethnical population [44,45]. In this study, the association between the variations of the *eNOS *gene (*G894T*) and the presence of nephropathy in SLE was significant, with the *eNOS 894T *allele play a significant protect role in the pathology of renal injury in SLE, and *eNOS 894G *allele in dominant genetic model were susceptibility gene for lupus nephropathy. However, no such association was found with the *eNOS T-786C*.

The above inconsistency could be due in part to the heterogeneity of the *ACE *gene and the *eNOS *gene among different ethnic groups [21,46], since it is often the case that the same SNP has different roles in different populations. Another reason may be the unclear stratification of the patients with the lupus nephropathy, such as first attack or recurrence and the age of onset and so on. Furthermore, environmental factors for cases and controls were not available in this study, which limited the analysis of the interaction between genetic factors and environmental factors, and further the estimation of adjusted ORs. Finally, the sample size for this study was relatively small due to the limited time of the collection of cases, the result of this study need validation.

Although some limitations do exist in this study, ours is an innovative study with some promising findings that contribute to the evaluation of genetic risk factors for lupus nephropathy. The clues provided in this study call for further research, other association studies, as well as functional studies are warranted to prove the association from multiple aspects. For the analysis of the association between these SNPs and the progression of this disease. Further studies, with larger sample sizes, are necessary to determine the contribution of these alleles to lupus nephropathy progression.

## Conclusions

Our study shows that two SNPs (*-5466C *and *2350G*) of the *ACE *gene and the one SNP (*G894T*) of the *eNOS *gene were associated with the susceptibility of lupus nephropathy. Five haplotypes (CTTCGA, ACTTAA, ACATGG, ACACGG and ATTCGA) were found to contribute to the risk of lupus nephropathy, suggesting an important role they played in the pathology of lupus nephropathy.

## Competing interests

The authors declare that they have no competing interests.

## Authors' contributions

XL participated in the molecular study and drafted the manuscript. JA performed the primer design and participated in the molecular study. RG and HL performed the statistical analysis. PL and ZJ participated in the statistical analysis and performed haplotype reconstruction. YL and YZ collected the patient and control blood samples. YZH, XJ and FL participated in its design and coordination and helped to draft the manuscript. All authors contributed to data interpretation and manuscript revisions, and all approved the final manuscript.

## Pre-publication history

The pre-publication history for this paper can be accessed here:

http://www.biomedcentral.com/1471-2350/11/94/prepub
